# Supplementary material for: Defining Microbial Community Functions in Chronic Human Infection with Metatranscriptomics
Source: bioRxiv. 2023 Jun 7:2023.06.06.543868. Preprint. [Version 2] doi: 10.1101/2023.06.06.543868 (PMC10274682; doi:10.1101/2023.06.06.543868)
Supplement: Supplement 1 [file media-1.pdf]

Supplementary Material

Materials and Methods

Dataset Collection: The datasets used for this study were identified by body part or tissue being affected in samples collected – chronic lower extremity ulcers for CW samples and sputum samples from people with CF, presence of original metatranscriptomic data, presence of comorbidities in patients and treatment course.

Community Composition Analysis: We reduced our Metaphlan4 output data to genera and species that had at least one percent abundance prevalence in at least 3 samples.

Functional Profiling: Two bioinformatic tools were used for functional profiling. The Simple Annotation of Metatranscriptomes by Sequence Analysis tool **(**SAMSA2) identified 4527 level 4 enzyme classes and the DIAMOND_analsys_counter.py scripts were used to aggregate the outputs which were eventually exported to R studio for statistical analysis. The diversity_stats.R script of the SAMSA2 package was used to compute the mean Shannon and Simpson diversity indices for the two infection communities and the diversity_gaphs.R script was used to plot the graphs of these. We performed differentially expression analysis of the features in the both communities using the run_DESeq_stats.R script. The HMP Unified Metabolic Analysis Network (HUMAnN3) tool, identified 594,273 UniRef protein families which were further regrouped to 2459 unique ECs using the humann_regroup_table command and exported to Rstudio for differential expression analysis.

Statistical Analyses:

For the HUMANn3 functional analyses, we performed differential expression analysis using DESeq2 1.38.3 and MaAsLin2 1.12.0 in R studio.

Figures

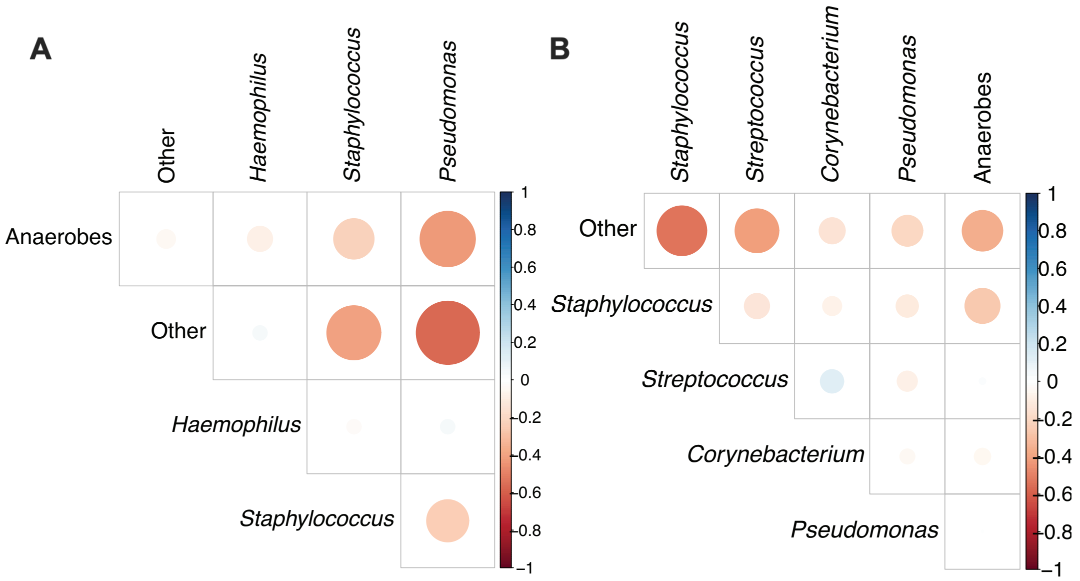

Figure S1: Co-occurrence of obligate anaerobes and traditional pathogens in CF and CW samples. **A)** Obligate anaerobes (*Prevotella*, *Actinomyces*, *Veillonella* & *Fusobacterium*) have negative correlation with CF pathogens (*Pseudomonas*, *Staphylococcus*, & *Haemophilus*). **B)** Obligate anaerobes (*Finegoldia, Anaerococcus*, *Peptoniphilus*, *Peptostreptococcus*, *Parvimonas* and *Peptococcus*) have negative correlation with CW pathogens (*Staphylococcus*, *Streptococcus*, *Pseudomonas*, and *Corynebacterium*). **A&B** show the Pearson correlation coefficients between the sum of the relative abundances of the anaerobes and each indicated pathogen. Other bacterial genera present in the community are grouped as other.

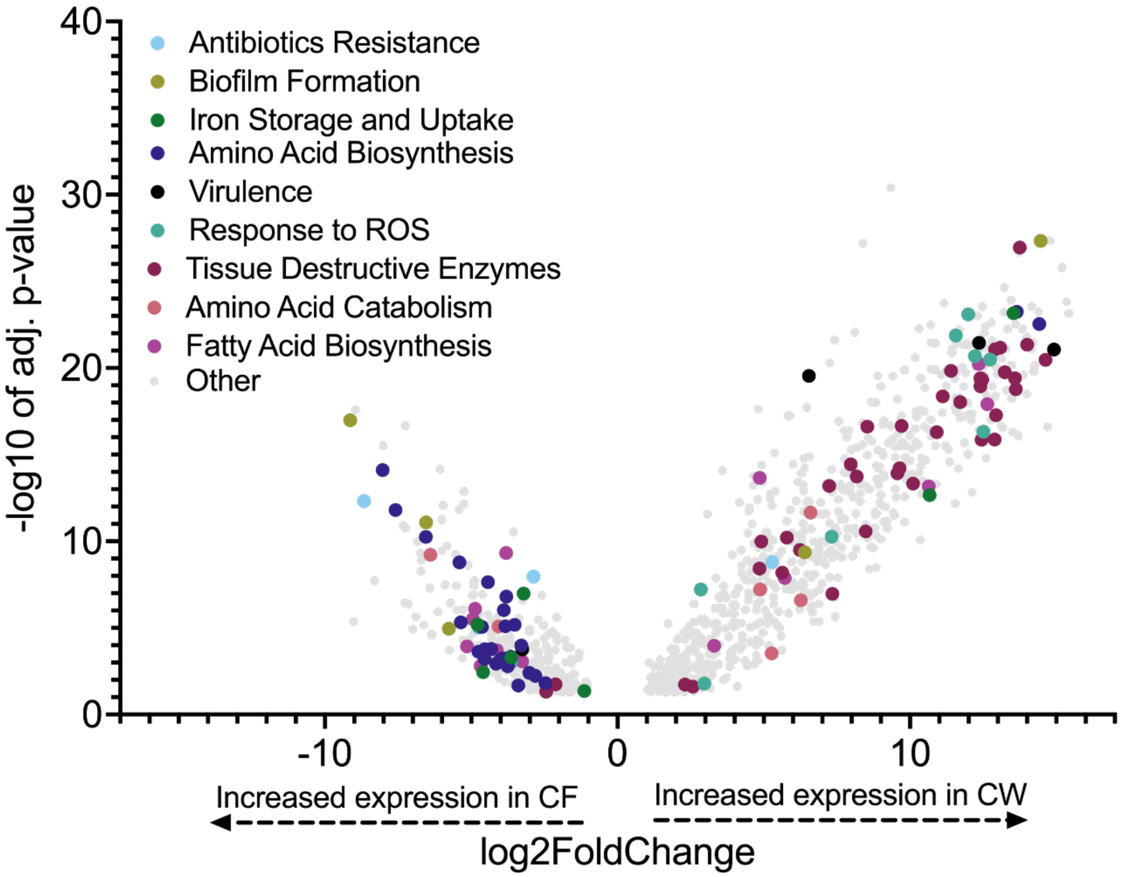

Figure S2: Volcano plot to highlight differentially expressed functions in both infection communities as identified by HUMANn3. 42.98% of the functions were differentially expressed (adjusted p-value <0.05, log2FoldChange > 1).

Supplementary Datasets

Dataset S1: Detailed metadata on all samples used for the study.

Dataset S2: Functional analysis data. Sheet 1 - highlighted conserved functions identified with HUMANn3 and SAMSA2 and bacterial contribution to these functions. Sheet 2 - highlighted differentially expressed functions identified with HUMANn3 and SAMSA2 and bacterial contribution to these functions. Sheet 3 - all differentially expressed functions obtained from HUMANn3. Sheet 4 - all differentially expressed functions obtained from SAMSA2.

Dataset S3: Pathway analysis data. Sheet 1- all pathway analysis data as identified by HUMANn3. Sheet 2 - all differentially expressed pathways obtained from HUMANn3.
